# Long Noncoding Ribonucleic Acid *SNHG18* Promotes Glioma Cell Motility *via* Disruption of α-Enolase Nucleocytoplasmic Transport

**DOI:** 10.3389/fgene.2019.01140

**Published:** 2019-11-13

**Authors:** Rong Zheng, Qiwei Yao, XiaoBo Li, Benhua Xu

**Affiliations:** ^1^Department of Radiation Oncology, Fujian Medical University Union Hospital, Fuzhou, China; ^2^Department of Radiation Oncology, Fujian Cancer Hospital, Fujian Medical University Cancer Hospital, Fuzhou, China; ^3^College of Medical Technology and Engineering, Fujian Medical University, Fuzhou, China

**Keywords:** Glioma, long noncoding ribonucleic acids, SNHG18, α-enolase, cell migration

## Abstract

Glioma is a common malignancy with poor prognosis. Recent evidence suggests that the pathogenesis and progression of glioma involve long noncoding RNAs (lncRNAs). Previously, we showed that glioma cell radioresistance was enhanced by lncRNA *SNHG18 in vitro* and *in vivo*. In the present study, we showed that *SNHG18* promoted the invasion and migration of glioma cells. *SNHG18* was demonstrated to regulate the progression of epithelial-mesenchymal transition and cytoskeleton remodeling, thereby affecting cell motility. Furthermore, the promotion of invasion evoked by *SNHG18* overexpression could be rescued by α-enolase (ENO1) deletion. Moreover, rather than altering ENO1 expression, *SNHG18* suppressed its nucleocytoplasmic transport by directly combining with ENO1 in glioma cells. The results suggested that *SNHG18* inhibited the nucleocytoplasmic transport of ENO1 to promote cell motility. The results reveal the mechanism by which this lncRNA affects tumorigenesis and metastasis, forming the basis for further research that will lead to novel strategies to treat glioma.

## Introduction

Glioma is the most common adult malignant brain tumor; however, the clinical prognosis of high-grade glioma is extremely poor, with a low 5-year survival rate (Jansen et al., 2010). High-grade glioma shows rapid progression and high invasiveness (Lefranc et al., 2005; Stupp et al., 2009). Current treatments are ineffective, partly because of the high invasiveness of brain tumor cells. In addition, tumor recurrence occurs as a result of the inevitable infiltration of the remaining tumor cells into the surrounding normal brain tissue (Rao, 2003). Local invasion is a major cause of mortality; therefore, improving our understanding of the molecular mechanisms involved in the invasiveness of glioma is vital.

Long noncoding RNAs (lncRNAs) are RNA molecules of greater than 200 nucleotides in length that do not encode proteins (Wang and Chang, 2011; Tay et al., 2014). Recent studies have shown that the development and progression of many cancers are regulated by certain lncRNAs (Yang et al., 2014). Growing evidence indicates that in the tumorigenesis of glioma, lncRNAs may have tumor suppressor or carcinogenic effects (Zhang et al., 2012). For example, lncRNA *H19* expression is markedly induced in glioma and correlates positively with the glioma grade, and its expression is required for tumor invasion and progression (Jiang et al., 2016). LncRNA *HOTAIR* is negatively associated with glioma prognosis *via* its promotion of the invasion and growth of glioma cells (Zhang et al., 2013; Zhou et al., 2015). Thus, lncRNAs represent good candidate biomarkers and potential therapeutic targets in glioma.

The enzyme α-enolase (ENO1), which was originally identified as a vital catalysis-associated protein, is an isoform of enolase that is found in almost all adult mammalian tissues (Pancholi, 2001). Decades of research have shown that in addition to its normal glycolysis function, ENO1 is involved in several key biological processes in glioma and other cancers, including proliferation, migration, and invasion (Capello et al., 2011; Song et al., 2014; Chen et al., 2018). Depending on its location in the tumor, ENO1 can exert multiple functions (Diaz-Ramos et al., 2012). It acts as a cell surface receptor for plasminogen and as a cytoskeletal reorganization regulator in the cytoplasm, promoting the invasion of metastatic cancer (Capello et al., 2011; Ceruti et al., 2013; Song et al., 2014; Principe et al., 2017; Yu et al., 2018).

Previously, we observed upregulation of small nucleolar RNA host gene 18 (*SNHG18*; GenBank Accession no. NR_045196) in glioma tissues. In addition, *SNHG18* expression showed an association with the clinical tumor grade and a negative correlation with mutation of isocitrate dehydrogenase 1 (IDH1). *SNHG18* could promote the radioresistance of glioma cells (Zheng et al., 2016). However, our previous study did not clarify the other functions of *SNHG18* in glioma in addition to radiosensitivity. In the present study, we further studied the biological functions associated with the migration and invasion of *SNHG18* and its underlying mechanisms. We also described the relevance of ENO1 in *SNHG18*-mediated glioma migration and investigated the underlying mechanisms. These results will contribute to identify potential candidates for targeted therapeutic interventions in glioma.

## Materials and Methods

### Cell Culture

The M059J and M059K cell lines were purchased from the ATCC (Rockville, MD, USA). U87 glioma cells were obtained from the Shanghai Institute of Cell Biology, Chinese Academy of sciences. The cells were maintained as described previously (Zheng et al., 2016).

### Quantitative Real-Time Reverse Transcription Polymerase Chain Reaction

The TRIzol Reagent (Invitrogen) was used to isolate total RNA from freshly cultured cells, following the manufacturer's instructions. Complementary DNA (CDNA) was synthesized using a PrimeScript ^®^ RT Reagent Kit (Takara, Dalian, China). PCR reactions were performed using a LightCycler 480 Real-Time PCR System (Roche, Stockholm, Sweden). The results were calculated as the ratio of the optical density (OD) value relative to that of *ACTB* (encoding β-actin). Primer sequences used for qRT-PCR were as follows: β-actin F: 5′-CCCTTTTTGTCCCCCAAC-3, ′ R: 5′-CTGGTCTCAAGTCAGTGTACAGGT-3′; *SNHG18* ′: 5′-TGTGGCAGCCCACTCTATTG-3, ′ R: 5′-TGGTGGACTT′GAGTGGAAGC-3′; ENO1 F: 5′-GATCTCTTCACCTCAAA′AGG-3, ′ R: 5′-TTCCATCCATCTCGATCATC-3. ′

### Construction of Glioma Cells Stably Overexpressing or Deleted for *SNHG18*

Cell clones stably overexpressing and deleted *SNHG18* were established as described previously (Zheng et al., 2016).

### Western Blotting

Cytoplasmic and nuclear proteins were extracted with an NEPER Nuclear and Cytoplasmic Extract Kit (Pierce, Rockford, IL, USA) according to the manufacturer's instructions. Aliquots of cell lysates were separated using a 12% sodium dodecyl sulfate (SDS)-polyacrylamide gel and then electroblotted onto nitrocellulose membranes (Bio-Rad), as previously described (Xie et al., 2014). The membranes were incubated with antibodies raised against ENO1 (#ab155102) and β-catenin (#ab32572) (both from Abcam, San Francisco, CA, USA), and those recognizing β-actin (#4970), N-cadherin (#13116), E-cadherin (#3195), snail family transcriptional repressor 2 (SLUG) (#9585), Vimentin (#5741), MMP-2 (#40994), MMP-9 (#13667), lamin A (#86846), and snail family transcriptional repressor (SNAIL) (#3879) (all from Cell Signaling Technology, Beverly, MA, USA) overnight, followed by the addition of horseradish peroxidase-linked anti-rabbit/mouse immunoglobulin G (IgG) (Abcam #ab205718/ab205719) and enhanced chemiluminescence visualization of the immunoreactive protein bands.

### Ribonucleic Acid Interference

RNA interference was performed as described previously (Zheng et al., 2016). A short interfering RNA (siRNA) targeting *ENO1* was obtained from GenePharma (Shanghai, China) and was transfected into cells using Lipofectamine 3000 (Invitrogen) following the manufacturer's instructions.

### Invasion Assay

Invasion assays were carried out using a Matrigel Invasion Chambers (BD Bioscience, Bedford, MA, USA) following the manufacturer's instructions. The chamber has two compartments divided by a polycarbonate filter (8-mm pore size). Transfected cells (3 ×10^5^ M059K and M059J cells, or 4 × 10^5^ U87 cells per 200 µl) in serum-free medium were placed in the upper chamber precoated with Matrigel. Complete medium (500 µl) containing 10% fetal bovine serum as a chemoattractant was placed into the lower chamber. The Transwell chambers were incubated for 24 h at 37°C. Thereafter, the filters were removed and the cells on them were fixed and then stained using 0.1% (vol/wt) crystal violet. The number of invading cells was counted from at least seven fields on three separate membranes using a light microscope (Olympus, Japan) with a 10× objective.

### Wound-Healing Assay

M059K, M059J, or U87 cells were grown in plates to form confluent monolayers, which were scratched using a pipette tip. The cell debris was removed and the width of the scratch was measured under an Olympus microscope (Tokyo, Japan) at 200× magnification. The cells were incubated in the serum-free medium for 24 h. Thereafter, healing was evaluated by measuring the width of the scratch under the microscope and comparing it with the original width.

### Immunocytochemistry and Confocal Microscopy

Cells were grown on confocal dishes (Corning Inc. Corning, NY, USA), washed, and fixed with 4% paraformaldehyde. The cells were then incubated with tetramethyl rhodamine iso-thiocyanate-conjugated phalloidin (Sigma, St. Louis, MO, USA) or an anti-ENO1 monoclonal antibody (Abcam #ab155102), followed by fluorescein isothiocyanate-conjugated anti-rabbit IgG (Abcam #ab150077). Nuclei were stained using 2-(4-amidinophenyl)-1H-indole-6-carboxamidine (Invitrogen) and viewed using a fluorescence confocal microscope (OLYMPUS FV10i). Olympus FV10-ASW software was used to capture the confocal images.

### Ribonucleic Acid Pull-Down Assay

A TranscriptAid T7 High-Yield Transcription Kit was used to transcribe *SNHG18* fragments, and antisense RNA from vector pcDNA3.1-6195. A Pierce™ magnetic RNA–protein pull-down kit was used for the RNA–protein pull-down experiments, which performed according to the manufacturer's protocols. The proteins retrieved were subjected to 12% SDS-polyacrylamide gel electrophoresis (PAGE) separation and a Pierce^®^ Silver Stain Kit was used. The specific silver-stained bands were excised and subjected to mass spectrometry analysis using the Mascot software. The experiments described in this section used kits obtained from Thermo Fisher Scientific (Waltham, MA, USA).

### Ribonucleic Acid Immunoprecipitation Assay

A Magna RIP™ RNA-Binding Protein Immunoprecipitation Kit (Millipore, Danvers, MA, USA) was used to perform the RNA immunoprecipitation (RIP) assay according to the manufacturer's instructions. For the RIP assay, the anti-ENO1 antibody was provided by Abcam (# ab155102). Reverse transcription PCR was used to determine whether *SNHG18* was present in the co-precipitated RNA.

### Statistics

All experiments were conducted in triplicate, unless otherwise stated. SPSS software (IBM Corp., Armonk, NY, USA) was used to perform the statistical analyses. Student's t-test analyzed the data for statistical significance. The statistical evaluations are shown as the mean ± standard deviation; a p-value <0.05 indicated statistical significance.

## Results

### SNHG18 Expression Promotes Invasive and Migratory Ability in Glioma Cells

**Figure 1:**
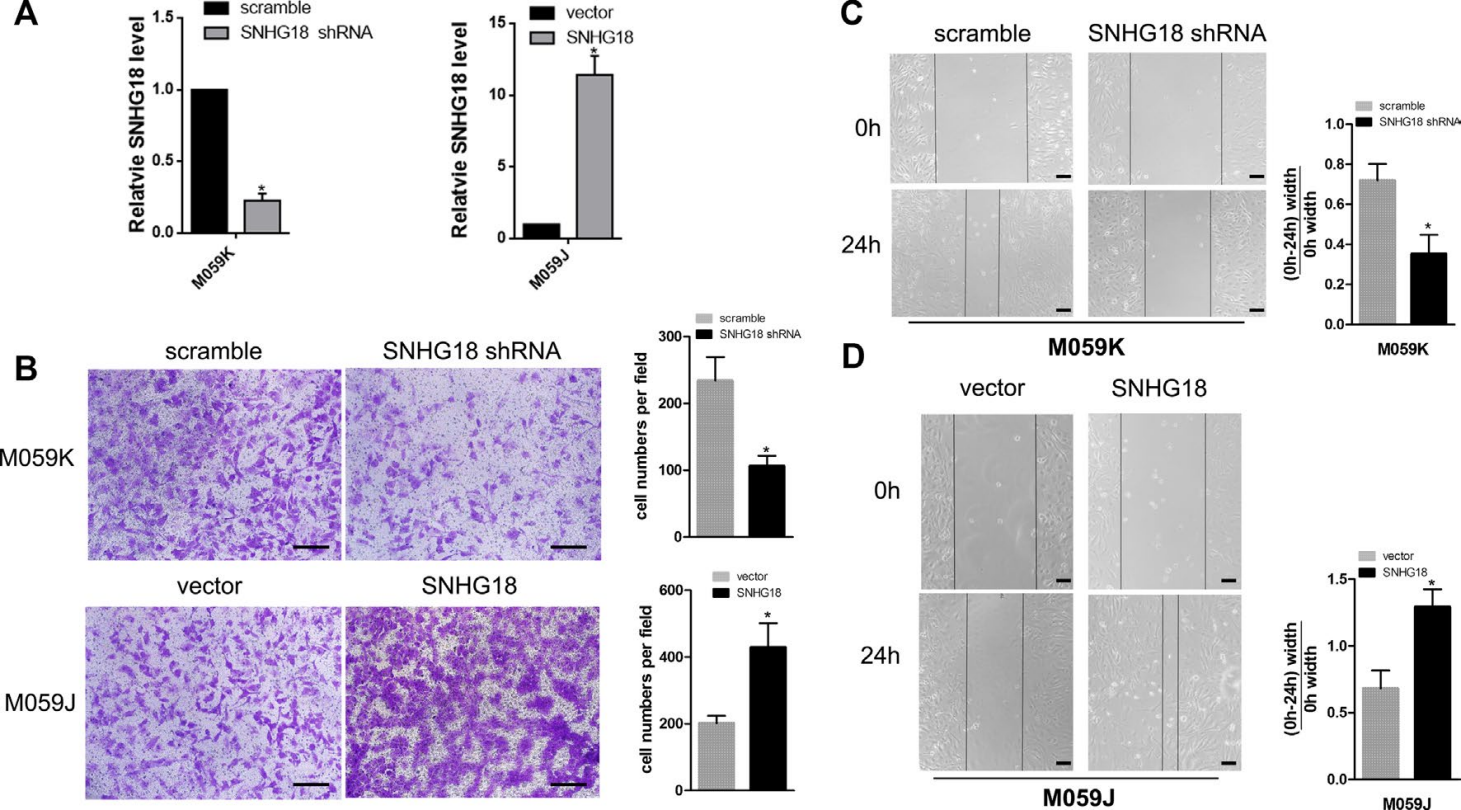
*SNHG18* promotes migration and invasion of glioma cells **(A)** The expression of *SNHG18* in M059K cells silenced for *SNHG18*, in M059J cells overexpressing *SNHG18*, and in their control groups was assessed using quantitative real-time reverse transcription polymerase chain reaction (mean ± SD, n = 3; *P < 0.05, Student's t-test). **(B)** Results from Transwell invasion assays of cells silenced for *SNHG18*, in M059J cells overexpressing *SNHG18*, and in their control groups, scale bar = 50 μm (mean ± SD, n = 3, *P < 0.05, Student's t-test). The results of wound-healing assays in M059K cells **(C)** silenced for *SNHG18*, in M059J cells **(D)** overexpressing SNHG18, and in their control groups. Scale bar = 200 μm (mean ± SD, n = 3, *P < 0.05, Student's t-test).

First, M059K cells stably silenced for *SNHG18* and M059J cells stably overexpressing *SNHG18* were constructed in our previous study (Zheng et al., 2016) (**Figure 1A**). Transwell invasion assays showed a significant decrease in invasion of the M059K-SNHG18 short hairpin RNA (shRNA) (*SNHG18* silenced) cells compared with that in the control group (106.2 ± 15.4 cells and 238.8 ± 35.6 cells, **Figure 1B**, P < 0.05, upper panels). Compared with the control group (201.2 ± 22.4 cells), the M059J-*SNHG18* (*SNHG18* overexpressing) cells showed significantly increased migratory ability (429.1 ± 72.8 cells, **Figure 1B**, P < 0.05, bottom panels). The effect of *SNHG18* on the glioma cell invasion was determined using a wound-healing assay. M059K cells transfected with the *SNHG18* shRNA showed significantly reduced migration compared with cells transfected with the scrambled shRNA control (**Figure 1C**, P < 0.05). By contrast, M059J cells overexpressing *SNHG18* displayed an increased migration ability, resulting in nearly complete closure of the wound after 24 h compared with that in the control group (**Figure 1C**, P < 0.05).

### *SNHG18* Suppresses Nucleocytoplasmic Transport of α-Enolase by Directly Combining With the A-Enolase Protein

**Figure 2:**
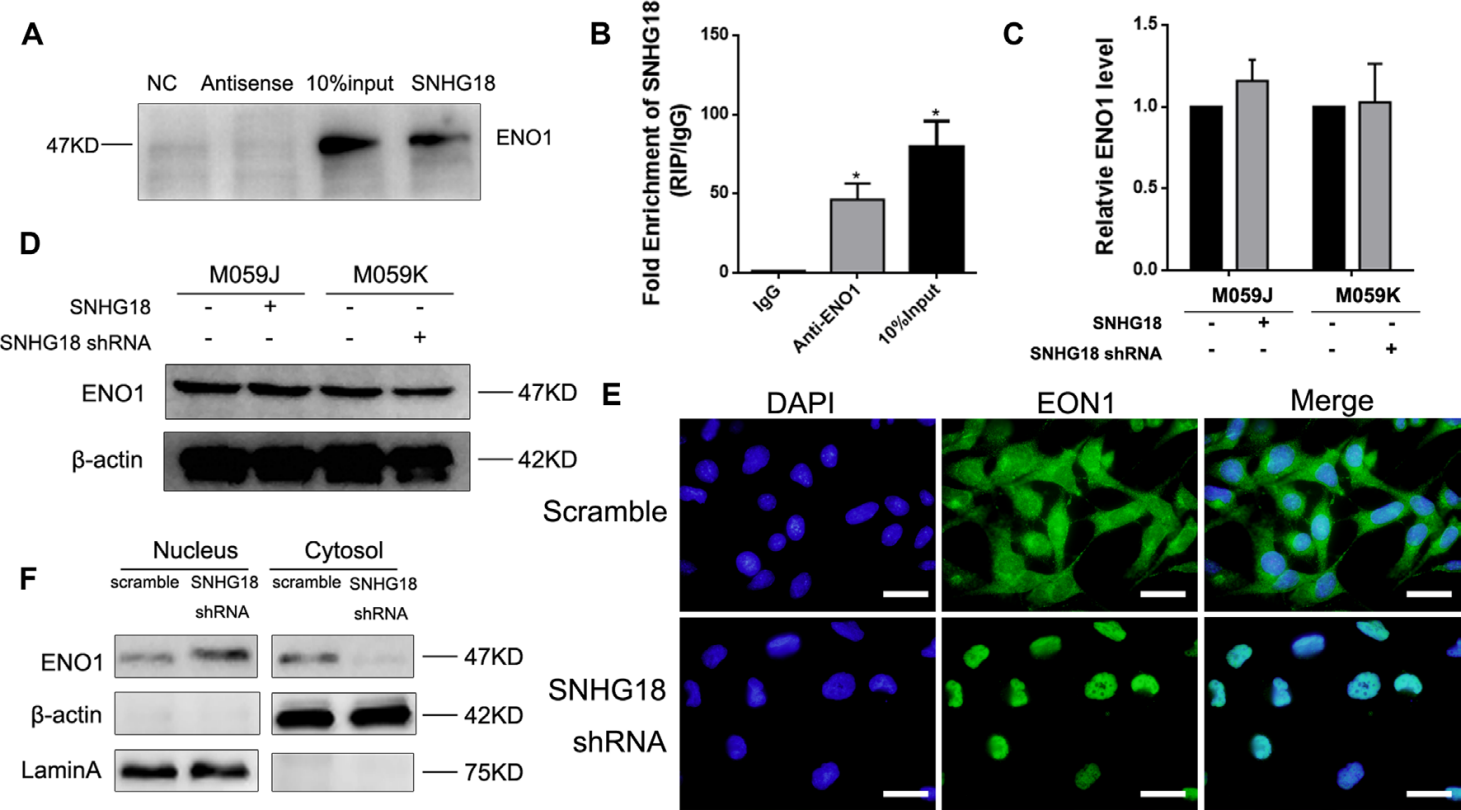
**(A)** The results of RNA pulldown using *SNHG18*, its antisense RNA, and a negative control RNA, as analyzed using western blotting for α-enolase (ENO1). The input was the total protein used for RNA pulldown (mean ± SD, n = 3, *P < 0.05, Student's t-test). **(B)** The results of RIP assays assessed using real-time reverse transcription polymerase chain reaction (RT-PCR) for *SNHG18*. Quantitative RT-PCR (mean ± SD, n = 3, *P < 0.05, Student's t-test) **(C)** and western blotting **(D)** analysis of ENO1 expression in M059K cells silenced for *SNHG18*, in M059J cells overexpressing *SNHG18*, and in their control groups (mean ± SD, n = 3; *P < 0.05, Student's t-test). **(E)** M059K cells silenced for *SNHG18* stained for nuclei [2-(4-amidinophenyl)-1H-indole-6-carboxamidine, blue fluorescence], and ENO1 (anti-ENO1 monoclonal antibody followed by fluorescein isothiocyanate-conjugated anti-rabbit immunoglobulin G, green fluorescence) (scale bar = 10 μm). **(F)** ENO1 expression in nuclear or cytoplasm extracts by western blotting.

**Figure 3:**
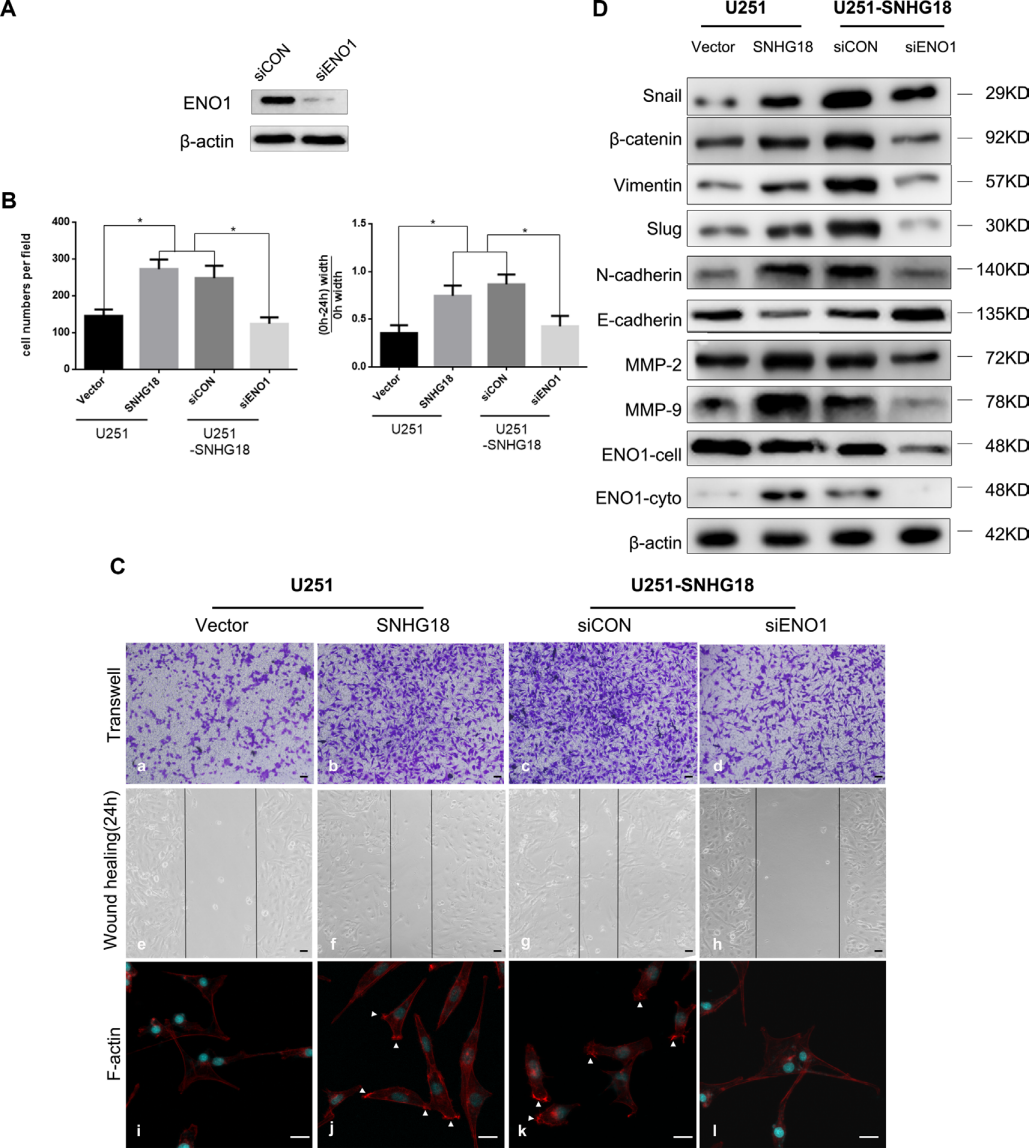
**(A)** Western blotting detection of the level of α-enolase (ENO1) in U251-*SNHG18* cells transfected with an short interfering RNA (siRNA) targeting ENO1 (siENO1). **(B)** Quantitative analysis of Transwell invasion and wound-healing assays in *SNHG18* overexpressed U251 cells treated with siENO1 and their control groups (mean ± SD, n = 3, *P < 0.05, Student's t-test). **(C)** Transwell invasion (Panel a-d) and wound-healing assays (Panel e-h), and immunofluorescence (Panel i-l) were performed in *SNHG18* overexpressed U251 cells treated with siENO1 and their control groups. Panel i-l: Tetramethyl rhodamine iso-thiocyanate-conjugated phalloidin was used to detect the distribution of F-actin (red). The nuclei are stained blue with 2-(4-amidinophenyl)-1H-indole-6-carboxamidine. Arrows show clustering of F-actin signals (Panel a-h: scale bar = 200 μm, Panel i-l: scale bar = 10 μm). **(D)** Western blotting to detect the levels of β-catenin, SNAIL, Vimentin, N-cadherin, SLUG, E-cadherin, MMP-2, MMP-9, and ENO1-total and ENO1-cyto (ENO1 in total cellular or cytoplasm) in U251-*SNHG18* cells transfected with the siRNA targeting ENO1 (siENO1).

Evidence shows that a lncRNA's regulatory mechanism correlates strongly with its location (Wang et al., 2011). We confirmed previously that *SNHG18* is located predominantly in the nucleus (Zheng et al., 2016). To determine whether *SNHG18*'s function involves interacting with a specific target, an RNA pull-down assay was performed, followed by mass spectrometry analysis to identify the proteins associated with *SNHG18*. The results indicated that ENO1 might be specifically associated with *SNHG18* (**Table 1**), which was subsequently confirmed by western blotting analysis (**Figure 2A**). RNA immunoprecipitation assays in M059K cellular extracts were then performed to confirm the association between *SNHG18* and ENO1. The RIP results showed a significantly higher enrichment of *SNHG18* using the anti-ENO1 antibody than was obtained using IgG (**Figure 2B**). These results showed that *SNHG18* could bind to ENO1 *in vitro*. Next we assessed the expression of ENO1 using qRT-PCR and western blotting (**Figures 2C, D**). ENO1 mRNA or protein levels did not change significantly after *SNHG18* deletion or overexpression. However, the translocation of ENO1 from the nucleus to the cytoplasm was clearly affected when *SNHG18* was silenced in M059K cells. The green fluorescence representing ENO1 protein in M059K cells was observed both in the nucleus and cytoplasm (**Figure 2E**, upper panel); however, in *SNHG18* silenced cells, no green fluorescence was observed in the cytoplasm (**Figure 2E**, lower panel), which was subsequently confirmed by western blotting analysis. In U251 cells, overexpressed *SNHG18* also did not change the expression of total cellular ENO1, but increased the content of ENO1 in the cytoplasm (**Figure 3D**). Thus, the interaction between *SNHG18* and ENO1 probably regulates the nucleocytoplasmic transport of ENO1 in glioma cells.

**Table 1:** Top 10 proteins identified using in mass spectrometry analysis among those proteins pulled down using *SNHG18.*

Protein hits	Score	emPAI^1^
ENO1	337	1.41
PRDX1	127	1.33
YWHAZ	195	1.21
ACTA2	226	0.98
PHB2	133	0.94
PPIB	172	0.93
TCEA1	270	0.92
TMOD3	389	0.9
KRT77	453	0.86
LMNA	404	0.83

^1^emPAI, exponentially modified protein abundance index.

### Removal of α-Enolase Suppresses the Promotion of Invasion Induced by *SNHG18* Overexpression

To determine whether *SNHG18*'s glioma cell motility promotion ability involves ENO1, we utilized *SNHG18* overexpressed glioma U251 cells with simultaneous suppression of ENO1 using a specific siRNA, siENO1. Effective knockdown of ENO1 expression in U251 cells was demonstrated using western blotting analysis (**Figure 3A**). Furthermore, *SNHG18* overexpression in U251 cells resulted in similar upregulation of migration and invasion to that observed in M059J cells (**Figures 3B, C**-panels a/b/e/f, *P < 0.05). ENO1 expression inhibition using siENO1 rescued the *SNHG18* overexpression-mediated reduction in migration and invasion; however, this phenomenon was not observed in the scrambled siRNA group (**Figures 3B, C**-panels c/d/g/h, *P < 0.05). Thus, *SNHG18* overexpression's effects on glioma cells depended at least in part on ENO1.

### *SNHG18* Enhances Cell Motility by Regulating the Progression of Epithelial-Mesenchymal Transition and Cytoskeletal Remodeling Depends on α-Enolase's Subcellular Location

To further investigate the mechanism by which *SNHG18* regulates glioma invasion, the protein levels of EMT-associated factors were examined in U251 cells stably overexpressing *SNHG18*. Enhanced endogenous *SNHG18* expression resulted in increased levels of β-catenin, SNAIL, SLUG, Vimentin, N-cadherin, MMP-2, and MMP-9, whereas E-cadherin levels decreased (**Figure 3D**). These results were partially reversed by ENO1 suppression. ENO1 participates in remodeling of the cytoskeleton, which has a pivotal role in the regulation of cell motility (Principe et al., 2017) and cell migration. Therefore, we studied the effect of *SNHG18* overexpression on the organization and morphology of the actin cytoskeleton. Phalloidin immunofluorescence staining of cell edges in untreated U251 glioma cells showed low numbers of lamellipodia, which are important structures involved in cell migration (Kozma et al., 1996) (**Figure 3C**-panel i). Remarkably, in U251 cells overexpressing *SNHG18* lamellipodia were significant induced and we observed a further reduction in the formation of long protrusions (**Figure 3C**-panel j). Silencing of endogenous *ENO1* expression using the siRNA abolished the disruption of actin-based lamellipodia, which persisted in the cells treated with the scrambled siRNA (**Figure 3C**-panels k and l). In summary, *SNHG18* regulates the progression of EMT and the organization of the cytoskeleton into lamellipodia by repressing the nucleocytoplasmic transport of ENO1 in glioma cells.

## Discussion

The main challenge presented by glioma is that the malignant cells can invade the adjacent normal brain tissue. Glial-origin tumors comprise a core mass and a penumbra of invasive cells, with decreasing numbers of cells towards the periphery; however, invasive cells can still be detected up to several centimeters distal to the core area (Burger et al., 1983). Local invasion eventually leads to tumor recurrence, mainly adjacent to the tumor bed. Local invasion does not improve significantly when treated by radiation or chemotherapy (Gaspar et al., 1992). Thus, the invasion of glioma occurs *via* independent mechanisms that encourage tumors to spread *via* various anatomical and molecular structures. Cell motility might be the common factor of this biological behavior. Genes associated with motility were observed to be upregulated in gene-expression profiling experiments, and functional studies suggested that the aggressive phenotype of malignant glioma is contributed by cell motility (Giese et al., 2003). Thus, it remains important to study the regulatory mechanisms leading to the invasive behavior in glioma.

Glioma usually exhibits large areas of hypoxia, which have more glycolytic activities than normal brain tissues, particularly in glioblastoma multiforme (Pfeiffer et al., 2001; Charles et al., 2011). Glioblastoma cells show strong migration abilities under glycolytic conditions (Beckner et al., 2005). The glycolytic enzyme ENO1 is essential for energy generation and anabolic processes in glioblastomas (Wise and Thompson, 2010). The expression of ENO1 is higher in glioma samples than in normal brain tissues, and is located mainly in the cytoplasm of glioma tissues, with weak expression in the cytoplasm in normal brain tissues. Increased levels of ENO1 protein were proven to be an important predictor of poor prognosis in patients with glioma (Song et al., 2014). Convincing evidence shows that lower ENO1 expression results in reduced cell growth, migration, and invasion (Song et al., 2014; Chen et al., 2018). ENO1 has a multifunctional role in promoting the invasion of metastatic cancer, depending on its localization in the cytoplasm (Capello et al., 2011; Ceruti et al., 2013; Song et al., 2014; Principe et al., 2017; Yu et al., 2018). These data strongly suggested that the role of ENO1 in the glioma tumor invasion depends on its subcellular localization.

LncRNAs regulate glioma tumorigenesis and metastasis in a variety of ways (Kiang et al., 2015). Our recent work revealed that the expression of lncRNA *SNHG18* was related to the clinical tumor grade and showed a negative correlation with mutations of IDH1 in glioma. *SNHG18* had the ability to promote radioresistance by inhibiting semaphorin 5A (Zheng et al., 2016). However, besides radiosensitivity, the other functions of SNHG18 in glioma remain unclear. The present study showed that *SNHG18* promoted the invasive and migratory abilities of glioma cells. *SNHG18* promoted cell motility by regulating EMT progression and remodeling the cytoskeleton.

Next, we investigated the detailed mechanisms of the invasive function of *SNHG18*. The localization of lncRNAs can have a profound influence on their molecular function and mechanisms. Especially, lncRNAs can induce significant cancer phenotypes by interacting with cellular macromolecules, including DNA, proteins, and RNA (Alexander, 1974). LncRNAs located primarily in the nucleus are more likely to recruit proteins to interaction with them (Quinodoz and Guttman, 2014). *SNHG18* is located mainly in the nucleus; therefore, we speculated that it might promote invasive and migration *via* interactions with nuclear proteins. RNA pull-down followed by mass spectrometry showed that *SNHG18* interacted with ENO1. Though ENO1 did not affect the radiosensitivity significantly (**Supplementary Figure 1**), knockdown of ENO1 expression using a small interfering RNA significantly suppressed the invasion-promoting action induced by *SNHG18*-overexpression in glioma cells. However, *SNHG18* did not alter the ENO1 expression level, rather it suppressed the nucleocytoplasmic transport of ENO1 by directly combining with ENO1. The ability of *SNHG18* to regulate EMT progression and remodel the cytoskeleton could be rescued by ENO1 deletion. These finding implied that *SNHG18* might regulate cell motility by disrupting ENO1 nucleocytoplasmic transport.

Taken together, the results of the present study revealed that increased expression of lncRNA *SNHG18* promoted the invasive and migratory ability of glioma cells by regulating EMT progression and cytoskeleton remodeling. The ability of *SNHG18* to regulate glioma cell motility depends on disrupting ENO1 nucleocytoplasmic transport. The results will lead to the development of novel therapeutic strategies to prevent and treat glioblastoma. However, our study has limitations. For example, the binding sites of *SNHG18* and ENO1 were not identified, and the upstream mechanism regulating *SNHG18* expression in glioma is unclear.

## Data Availability Statement

The raw data supporting the conclusions of this manuscript will be made available by the authors, without undue reservation, to any qualified researcher.

## Author Contributions 

RZ, XL, and BX contributed to the conception and study design. RZ and QY performed the experiments. RZ, QY, and XL analyzed the data. RZ drafted the manuscript. All authors read and approved the final manuscript.

## Funding

This study was supported by National Natural Science Foundation of China (Grant numbers: 81703041, 81803037), Fujian provincial health and family planning research talent training program (Grant number: 2017-ZQN-30) and Joint Funds for the innovation of science and Technology, Fujian province (Grant number: 2017Y9050).

## Conflict of Interest

The authors declare that the research was conducted in the absence of any commercial or financial relationships that could be construed as a potential conflict of interest.
