# Supplementary material for: Long Noncoding Ribonucleic Acid SNHG18 Promotes Glioma Cell Motility via Disruption of α-Enolase Nucleocytoplasmic Transport
Source: Front Genet. 2019 Nov 13;10:1140. doi: 10.3389/fgene.2019.01140 (PMC6865306; doi:10.3389/fgene.2019.01140)

SUPPLEMENTARY MATERIALS

Colony formation assays

Colony formation assay was performed to determine the radiosensitivity of cells. Cells were plated in 6-well plates and then adhered overnight before been exposured to irradiation at the indicated doses using 6-MV x-rays generated by a linear accelerator (Varian 2300EX) at a dose rate of 5 Gy/ min. After incubated for 10 to 14 days, the cells were stained with 0.5% crystal violet in methanol. The colonies (a population of >50 cells) were then counted using microscopy. The survival data from different experiments were pooled, and the survival curves were fitted and analyzed using the linear-quadratic model.

Fig 1. Clonogenicity survival assay performed in U251-SNHG18 cells transfected with an siRNA targeting ENO1 (siENO1) and their control groups (mean ± SD, n = 3).


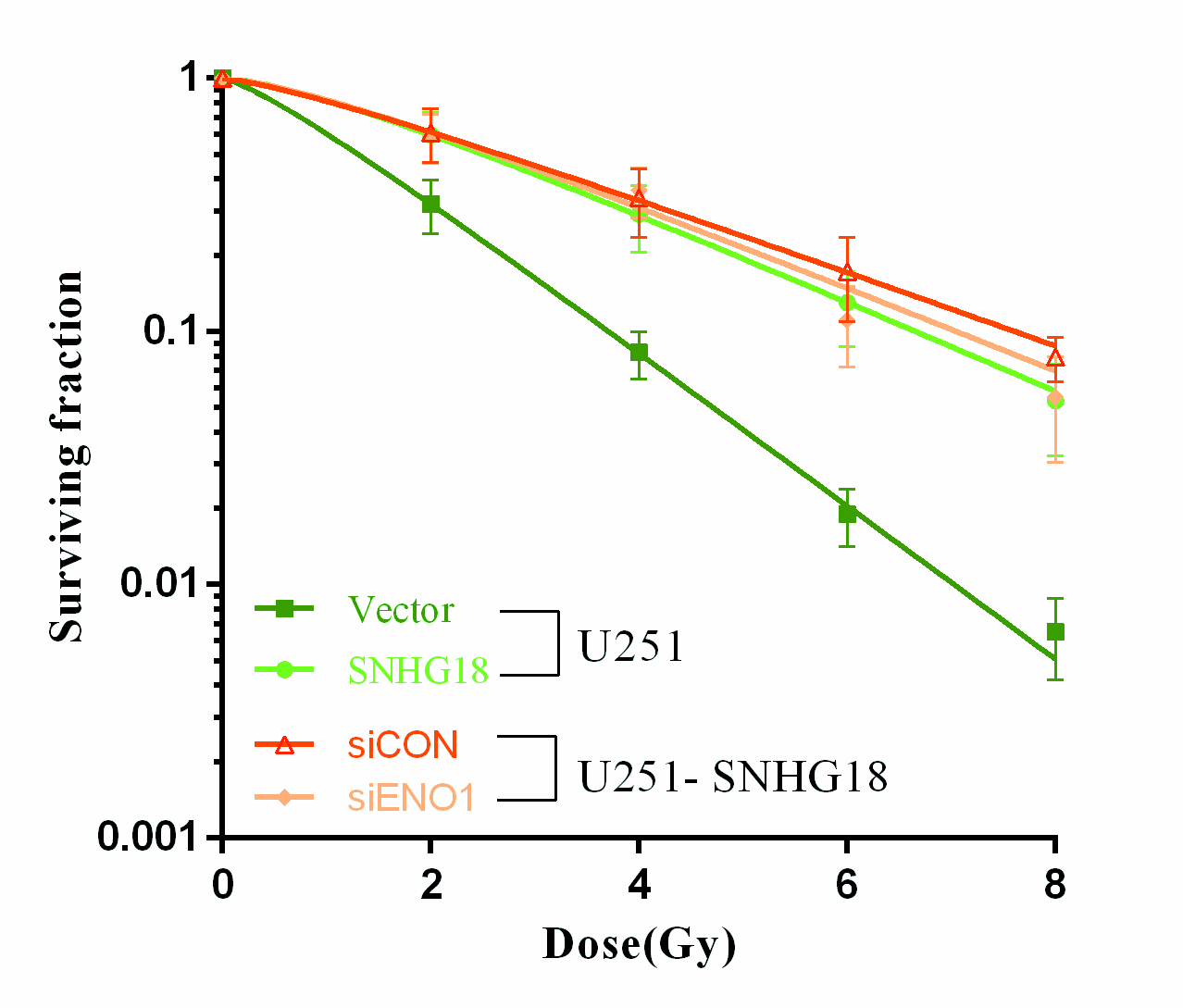

Supplement: Supplementary file 1 [file DataSheet_1.docx]
